# The Hawthorn (*Crataegus* spp.) Value Chain: An Integrated Analysis of Resource Availability, Phytochemical Characterization, and Therapeutic Applications

**DOI:** 10.3390/foods15010148

**Published:** 2026-01-02

**Authors:** Fengjin Zheng, Jing Chen, Yuan Tan, Xiaohua Dai, Xiangying Wei, Bo Lin, Krishan K. Verma, Gan-Lin Chen

**Affiliations:** 1 Institute of Agro-Products Processing Science and Technology, Guangxi Academy of Agricultural Sciences, Nanning 530007, China; linbo@gxaas.net; 2 Guangxi Subtropical Crops Research Institute, Guangxi Academy of Agricultural Sciences, Nanning 530001, China; jchen1030@gxaas.net (J.C.); xiaohuadai2023@163.com (X.D.); 15102932953@163.com (X.W.); 3 Key Laboratory of Quality and Safety Control for Subtropical Fruit and Vegetable, Ministry of Agriculture and Rural Affairs, Nanning 530001, China; 4 Guangxi Key Laboratory of Quality and Safety Control for Subtropical Fruits, Nanning 530001, China; 5 College of Agriculture, Guangxi University, Nanning 530004, China; 18373438400@163.com; 6 Sugarcane Research Institute, Guangxi Academy of Agricultural Sciences, Nanning 530007, China; drvermakishan@gmail.com

**Keywords:** large-fruited hawthorn, germplasm resources, functional effects, byproducts, bioactivity and pharmaceutical functions

## Abstract

Hawthorn is widely distributed across China, including Shanxi, Henan, Hebei, Shandong, and Shaanxi provinces. It is rich in functional components and nutritional elements, making it a crucial raw material for medicinal and food products. This review provides comprehensive information of the distribution of hawthorn germplasm resources in China and compares the differences in nutrient composition, chemical substances, and functional activities among different species. Furthermore, it offers a statistical analysis of the diversified processing and applications of hawthorn in China. Finally, the review identifies current challenges in the agro-food industries and states the future outlook of the industry. By systematically integrating research findings into a comprehensive “resource–characterization–application” framework, the study addresses the current fragmentation and lack of systematic organization in hawthorn research. It seeks to provide a scientific basis for directional breeding, strategic planning of production areas, precise product development, and high-quality development of the hawthorn industry in years to come.

## 1. Introduction

Hawthorn (*Crataegus pinnatifida* Bunge) has cultivated approximately 1000 varieties worldwide, primarily distributed across the Northern Hemisphere. Among these, China stands as a significant region with a long history of hawthorn cultivation. China possesses relatively abundant hawthorn resources. A survey of hawthorn resources proposed the classification and grouping of hawthorn species, identifying twenty-three species and six varieties [1]. Its cultivation area spans the entire country. The botanical characteristics among different hawthorn varieties vary, primarily influenced by the combination of atmospheric variables and human factors.

It is well known for its distinctive flavor, major nutrients, and bioactive compounds. In addition to standard macronutrients and minerals, hawthorn is rich in vitamin C, flavonoids, organic acids, and polyphenols. The advancement of food processing technologies has further popularized hawthorn as a widely consumed food product. Nationally developed hawthorn products include popular snacks like candied haws, cakes, canned goods, wine, vinegar, and slices, as well as healthy foods. The 2020 edition of the *Pharmacopeia of the People’s Republic of China* (Part 1) outlines the hawthorn fruit’s functions, which include aiding digestion, strengthening the stomach, promoting blood circulation, and reducing blood pressure and lipids. It is also beneficial for indigestion, diarrhea, abdominal pain, amenorrhea, postpartum complications, specific pains, and hyperlipidemia conditions.

Nowadays, significant progress has been made in research works on hawthorn in various fields, such as botany, chemistry, pharmacology, clinical medicine, food, and cultivation. Hawthorn boasts an abundant variety of resources. The search conducted in major databases, i.e., CNKI, Web of Science, PubMed, and Google Scholar, using “hawthorn” as the subject or keyword revealed that the main cultivated hawthorns in China include hawthorn, North hawthorn (North Crataegus, Bei shanzha), South hawthorn (South Crataegus, Nan Shanzha), large-fruited hawthorn, and Guang hawthorn (Guang shanzha, also called Cantonese Crataegus) [2]. However, a detailed analysis of the search results reveals that the current reviews on hawthorn tend to focus on relatively limited topics, such as hawthorn’s botanical taxonomy, processing techniques, and extraction of bioactive compounds. This review aims to provide a comprehensive overview of the Chinese hawthorn value chain by synthesizing information on germplasm resources, bioactive compounds and efficacy, processing applications, and other aspects, enabling readers to access information more efficiently.

## 2. Classification and Evolution of Hawthorn

**Figure 1:**
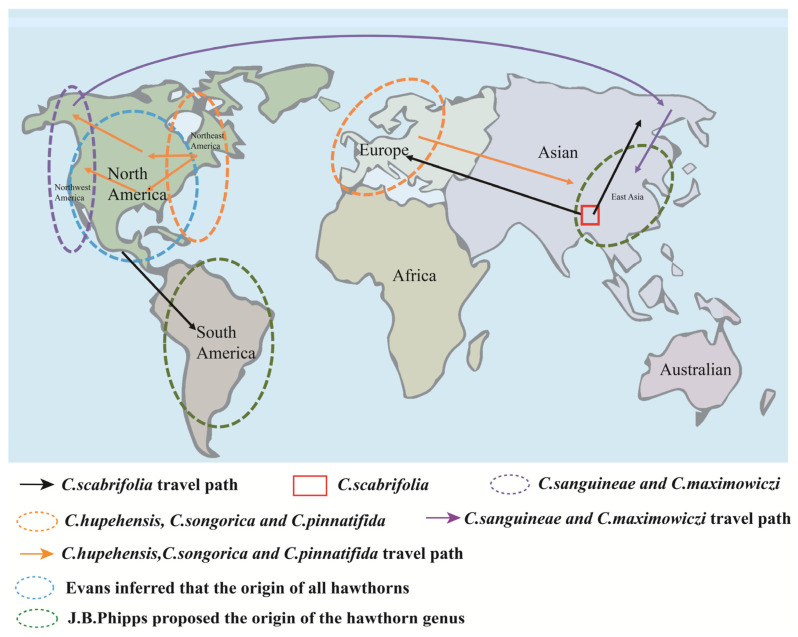
Worldwide distribution of hawthorn cultivation.

Hawthorn (*C. pinnatifida* Bunge) belongs to the genus *Crataegus* within the subfamily *Maloideae* of the Rosaceae family. It is widely distributed across Asia, Europe, and the Americas, between latitudes 20° N and 60° N [3]. There is considerable disagreement among scholars regarding the global classification, distribution, and total number of the *Crataegus* species, with estimates ranging from 150 to 1200 (Figure 1). However, most Chinese plant taxonomists concur that the genus comprises approximately 1000 species.

Chinese hawthorn refers to several species, in addition to *Crataegus pinnatifida* (*Crataegus pinnatifida* Bunge var. major N. E. Brown; *Crataegus pinnatifida* Bge.), called North Crataegus (Bei Shanzha) and included in the pharmacopeia of the People’s Republic of China, South Crataegus (Nan shanzha) and Cantonese Crataegus (Guang shanzha) are commonly used in the Chinese market. In fact, there are two types of large-fruited hawthorn with the same name in China. One type of large-fruited hawthorn refers to a variety of plants in the Sect. *Pinnatifidae* of the *Crataegus* genus in the *Maloideae* subfamily of the Rosaceae family. This species is distinguished from the original by its larger fruit, which ripens to a red color.

**Figure 2:**
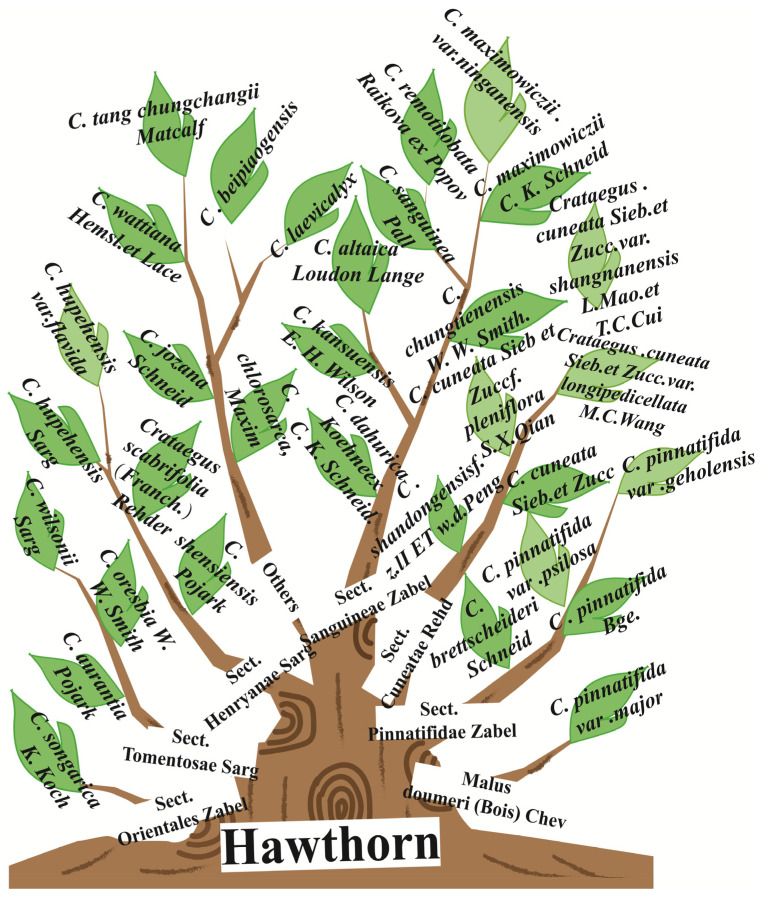
The classification of Chinese hawthorn germplasm resources.

Another type of large-fruited hawthorn is the *Malus doumeri* plant in the Sect. *Pinnatifidae* of the *Malus* Mill. genus in the *Maloideae* subfamily of the Rosaceae family, also known as Cantonese Crataegus (Guang shanzha). The mature fruit is green or greenish yellow. In China, hawthorn is primarily cultivated in Shandong, Shanxi, Henan, Hebei, Shan’xi, and Liaoning provinces for economic purposes mainly. In addition, Cantonese Crataegus fruit is large and shares similar pharmacological properties with the Chinese medicinal herb hawthorn, so it is often called “large-fruited hawthorn”. Cantonese Crataegus (Guang shanzha), including two closely related species from *Malus doumeri* (Bois) Chev. (locally called Taiwan crabapple) and *Malus leiocalyca* S.Z. Huang [4], are mainly distributed in the southwest of Guizhou, western and southwest of Jiangxi, southwest of Zhejiang, eastern and southeast of Hunan, Guangdong, Guangxi, and southeast of Yunnan in China and Taiwan. To attain a better understanding of Chinese hawthorn germplasm, resources are shown in Figure 2.

## 3. Distribution, Cultivation, and Characteristics of Hawthorn

*Crataegus* plant resources are relatively rich with widespread cultivation across China. Based on their natural geographical distribution, these plants can be classified into three groups, i.e., widespread, medium-range, and narrow-range species. Table 1 summarizes the survey of Chinese *Crataegus* resources and the classification systems proposed by Chinese scholars. As shown in Table 1, hawthorn resources are widely distributed and diverse. Currently, the primary hawthorn species used for medicinal purposes include the Northern hawthorn, which is approved for the use of food and medicine, as well as Southern hawthorn varieties. The southern varieties, namely the wild hawthorn (*C. cuneata* Sieb. et Zucc.) and the Cantonese Crataegus (Guang shanzha), are common in the regional market [5].

**Figure 3:**
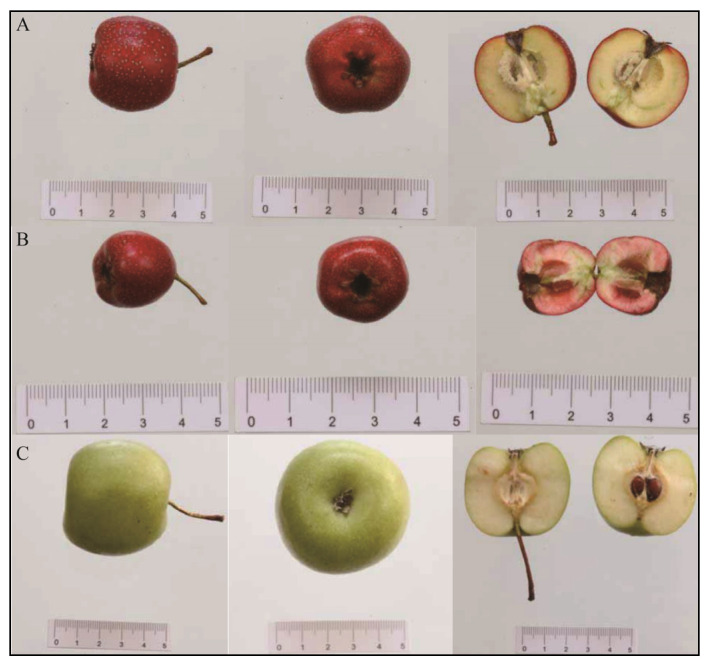
Comparative morphological analysis of different typical regional hawthorn varieties, i.e., North Crataegus (**A**), South Crataegus (**B**), and Cantonese Crataegus (**C**) [6].

Table 2 compares the traits of North Crataegus (Bei Shanzha), South Crataegus (Nan shanzha), and Cantonese Crataegus (Guang shanzha) cultivars, using carpel and seed characteristics as key identification criteria. Figure 3 shows the fruit shapes and cross-sections of different hawthorn types. The comparative analysis reveals the following orders, such as Southern < Northern < Cantonese for fruit size and flesh thickness, Northern < Southern < Cantonese for outer skin color, Northern < Cantonese < Southern for flesh color, and Northern < Southern < Cantonese for seed number.

## 4. Active Chemical Substances and Functional Effects

**Figure 4:**
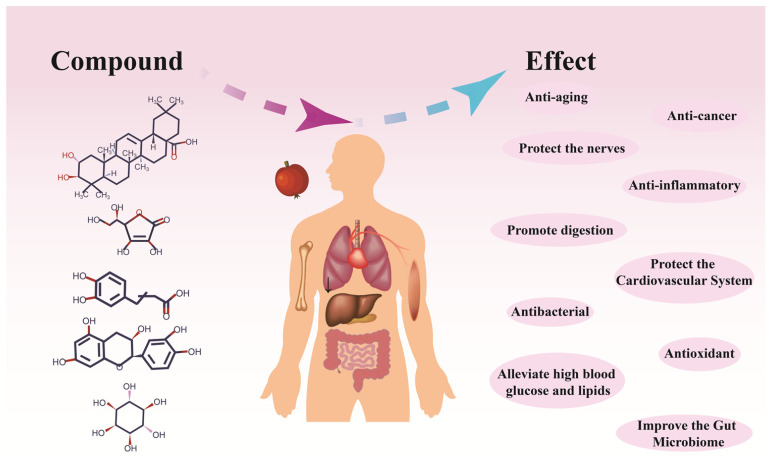
Hawthorn exhibits a range of pharmacological effects.

Hawthorn is rich in biologically active substances, such as sugar, flavonoids, phenols, terpenes, pectin, organic acids, etc. It is especially rich in pectin, which reaches 6.4%, and is ranked as the top of fruits; pectin exists in fruit tissues in different forms of protopectin, pectin, and pectinic acid [7]. Hawthorn has been used in traditional medicine since the ancient era. While its pharmacological effects were once unclear, they are now being elucidated through advances in research techniques (Figure 4).

### 4.1. Basic Nutrients

**Figure 5:**
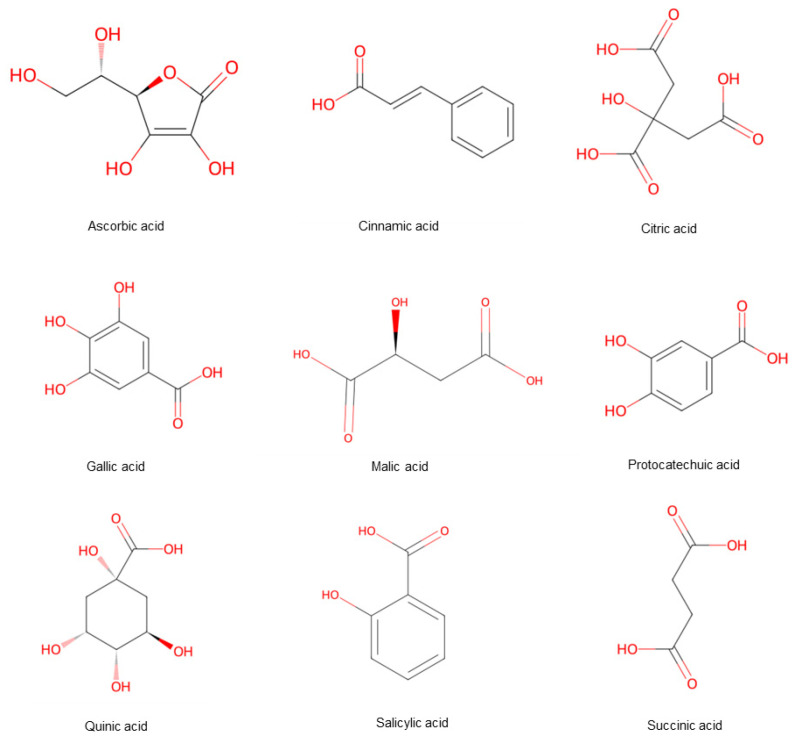
Hawthorn fruits comprise a variety of acid compounds. Note: Red color means oxygen-containing functional groups.

Hawthorn is notable for its high nutritional value, containing some of the highest levels of amino acids, proteins, trace elements, and vitamins among fruits, with particularly significant calcium (Ca^2+^) content [8]. Organic acids are widely distributed in the leaves, roots, and fruits of plants. Beyond modulating flavor, these organic acids have demonstrated physiological effects, including vasodilation (softening blood vessels), enhanced absorption of calcium and iron, and stimulation of digestive secretions. Consequently, they contribute to increased appetite, improved digestion, and relief from thirst and heat. A total of 76 organic acid metabolites were detected in hawthorn fruits (Figure 5).

Citric, malic, quinic, succinic, and ascorbic acids were identified as the predominant types [9]. Phenolic acids, which are derivatives of these organic acids, are notable for their potent antioxidant activity. Recent pharmacological studies have shown that phenolic acids possess various bioactivities, including antidiabetic effects. In vitro studies indicated that phenolic acids primarily exert antidiabetic effects by inhibiting the activity of α-glucosidase and α-amylase, thereby preventing blood glucose elevation [10]. South and North hawthorns contain gallic acid and p-hydroxybenzoic acid, while Guang hawthorns contain p-coumaric acid, ferulic acid, dihydrocaffeic acid, and genipic acid.

**Figure 6:**
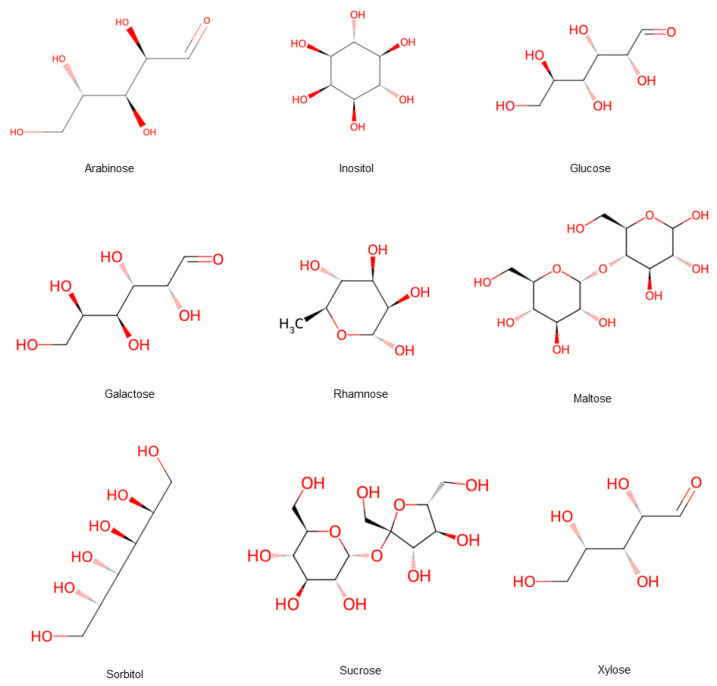
Hawthorn fruits contain a variety of sugar forms. Note: Red color means oxygen-containing functional groups.

The sugar in hawthorn primarily include maltose, sucrose, glucose, arabinose, galactose, rhamnose, xylose, sorbitol, and inositol, among other sugar and sugar alcohols [11] (Figure 6). It contains some functional sugars with multiple health benefits, such as regulating blood sugar levels. Northern hawthorn has higher total sugar content than its southern counterpart [12]. The sugar contains acidic polysaccharides and galacturonic acid, which form hawthorn pectin, which is a compound with diverse biological activities, such as antioxidant, anti-glycation, anti-tumor, and lipid-lowering effects, as well as prebiotic activity and heavy metal removal [7]. Hawthorn pectin is rich in polygalacturonic acid, which can be hydrolyzed by the enzyme polygalacturonase into pectin oligosaccharides. Experimental studies in SAMP8 mice have shown that these functional oligosaccharides exhibit senescence-delaying properties, and possess the primary mechanism involved in interfering with apoptosis induced by hydrogen peroxide, (H_2_O_2_), inhibiting the expression of key cell cycle factors, and stabilizing the cellular state [13]. Hawthorn is rich in pectin, a beneficial compound that has led to the development of various products like jelly, tea, and cake. As the seventh major nutrient, dietary fiber is crucial for preventing and mitigating conditions, such as constipation, hyperglycemia, colorectal cancer, and hypertension. Hawthorn pomace contains the highest levels of dietary fiber, and is therefore often considered a better source of non-traditional dietary fiber. The global annual disease burden indicates that mental disorders are a major public health problem. Consequently, advance treatment approaches like traditional herbal and dietary therapies are gaining attention [14].

### 4.2. Phenylpropanoids

**Figure 7:**
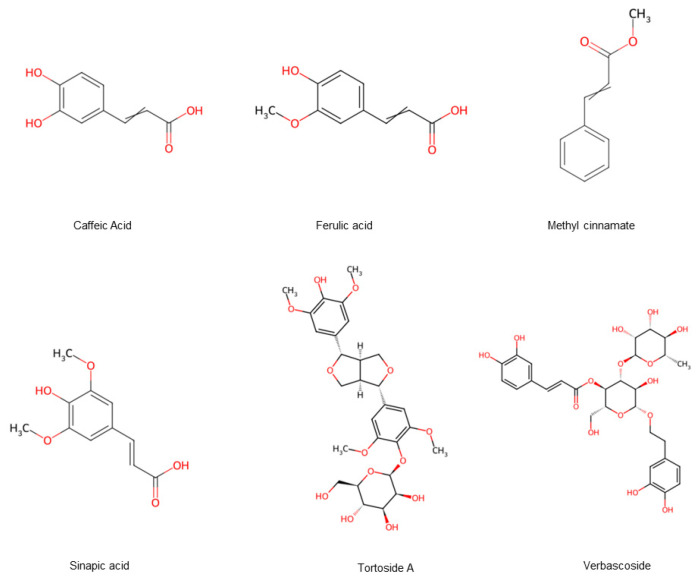
Hawthorn fruits contain phenylpropanoid compounds. Note: Red color means oxygen-containing functional groups.

Phenylpropanoids are a structurally rich and functionally diverse class of plant secondary metabolites. In hawthorn, 182 phenylpropanoids have been identified (Figure 7), which include simple phenylpropanoids (22 compounds), coumarins (2 compounds), and lignans (158 compounds) [15]. Phenylpropanoids exhibit diverse biological activities, including antioxidant, anti-inflammatory, and anti-tumor effects [16]. Studies have shown that more than 150 lignans, a subclass of phenylpropanoids isolated from hawthorn fruits, have demonstrated antioxidant, anti-inflammatory, and neuroprotective properties. The analysis of these compounds offers the potential for developing advance, cost-effective drugs with antioxidant and anti-inflammatory applications [17,18,19].

### 4.3. Phenolic Compounds

**Figure 8:**
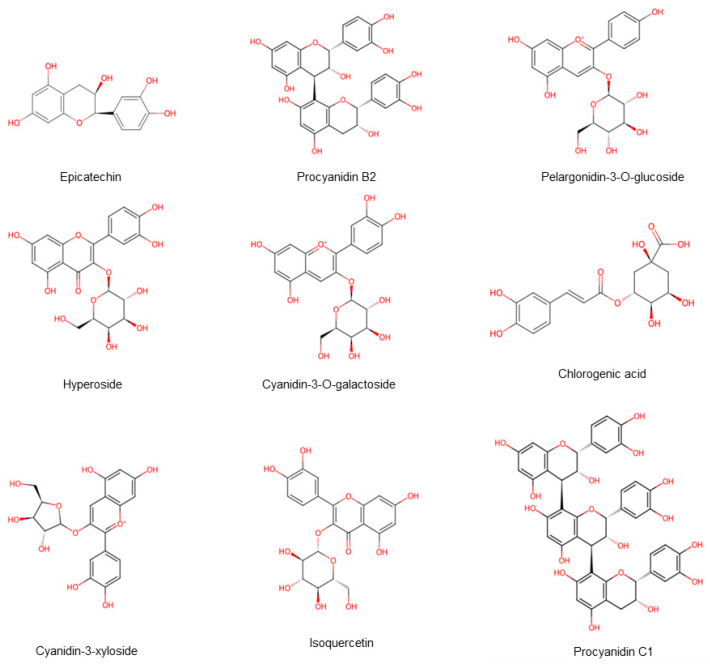
The availability of phenolic compounds in hawthorn fruits. Note: Red color means oxygen-containing functional groups.

Hawthorn ranked first among 30 anti-aging fruits, primarily due to its rich phenolic compounds. These include flavonoids, anthocyanins, and proanthocyanidins [20] (Figure 8). An acidic environment helps maintain the stability of phenolic compounds, and hawthorn is rich in organic acids, which provides a favorable condition for this stability [21]. Research findings indicated that the composition and concentration of phenolic compounds in plants are affected by numerous factors, including altitude, light exposure, soil conditions, and sampling location, and demonstrate positive correlation with ambient air temperature and sunlight [22]. This is exemplified by the phenolic profiles of different hawthorn species. The total phenolics in Northern hawthorn consist predominantly of chlorogenic acid, proanthocyanidin B2, and epicatechin, whereas in Guang hawthorn, they are primarily chlorogenic acid, phloridzin, and ursolic acid. In addition to its antioxidant activity, hawthorn is beneficial for managing a variety of conditions, including inflammation, cardiovascular disease, hypertension, diabetes, cancer, peptic ulcers, and microbial infections [23]. Researchers identified over 60 flavonoids in hawthorn [24], with the total flavonoid content varying between different varieties and plant parts, ranging from 2.27 to 17.40 mg/g [25]. Studies have found that the total flavonoid content in Southern hawthorn fruits is higher than Northern hawthorn.

For instance, in vitro studies have shown that hawthorn polyphenols aid digestion by influencing lipid and amino acid metabolism and modulating the intestinal microbiota [26,27,28]. Cardiovascular disease (CVD) is a leading cause of death worldwide and significant health risk in modern society. Early clinical studies indicated that hawthorn is effective in its prevention and treatment [29], as its fruits, leaves, and flowers exhibit antispasmodic, cardiotonic, antihypertensive, and anti-atherosclerotic properties. The antioxidant activity of hawthorn polyphenols and triterpenoids mediates their protective effects, which include inhibiting cell apoptosis [30], increasing myocardial glutathione peroxidase (GSH-Px) activity, reducing malondialdehyde (MDA) levels [31], and mitigating nitrosative stress and lipid peroxidation [32]. Furthermore, hawthorn polyphenols can also effectively mitigate other oxidative stress-related diseases, including diabetes [33], inflammation [34], depression [14], hyperlipidemia [35], and bacterial infections [36].

### 4.4. Terpenoids

**Figure 9:**
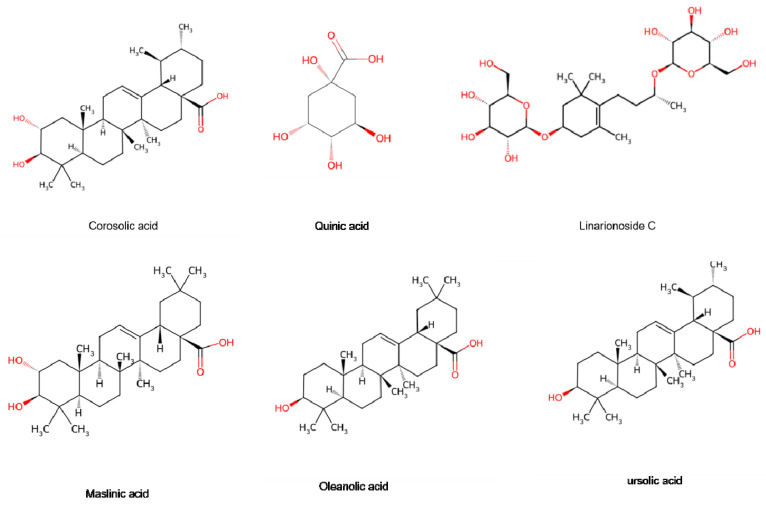
Terpenoids are present in the hawthorn fruits. Note: Red color means oxygen-containing functional groups.

Terpenoids are formed by the association of six isoprene units, primarily comprising monoterpenes, sesquiterpenes, triterpenes, and their glycosides. At present, 28 distinct terpenoids have been identified in the hawthorn [15] (Figure 9). Monoterpenes and sesquiterpenes are found in very low concentrations in the fruits but primarily located in the leaves [17]. These compounds serve as significant raw materials for the spice and pharmaceutical industries. As the main phytochemicals in Rosaceae fruits, triterpenoids, primarily triterpene acids, such as oleanolic acid, ursolic acid, and 2α-hydroxyursolic acid, are critical to hawthorn fruit quality [37]. The results of cellular experiments showed that ursolic acid, a major component of triterpenoids, exhibited strong inhibition of the growth of human cancer cells HepG2, MCF-7 and MDA-MB-231 [37]. These compounds impart potential tumor-relieving properties to hawthorn. We look forward to more scientific clinical studies confirming it as an ideal source of healthy foods with cancer-preventive effects.

The chemical profile of hawthorn varies significantly between northern and southern varieties. While Southern hawthorn has a lower concentration of ursolic acid, it is richer in corosolic, oleanolic, and quinic acids. These essential oil components are primarily derived from the mixture of terpenoids and phenylpropanoids [38]. There is little research on hawthorn essential oil and the existing research primarily focuses on the extraction and identification of components from its flowers [39,40]. This essential oil has a unique aroma and possesses antiviral and antibacterial properties, making it suitable for use in deterrents and attractants, and in the food, pharmaceutical, and perfume industries [41]. The global tumor burden is increasing, where high incidence and mortality rates present a severe public health challenge. Maslinic acid (MA), a natural pentacyclic triterpene acid found in hawthorn, exhibits anti-cancer effects by inhibiting tumor cell proliferation and inducing apoptosis. Cell-based studies have verified that MA can regulate different pathways, including the MAPK, OMA1, and MAPK/ERK signaling pathways, which regulate the growth of breast, nasopharyngeal, cervical, and human neuroblastoma cells [42,43,44]. Current cellular studies provide the basis for more clinical research focused on hawthorn actives.

### 4.5. Other Components

In addition to the aforementioned functional substances, a wide array of other compounds including nitrogen compounds, such as choline, acetylcholine, squalene, anthraquinones, cardiac glycosides, alkaloids, peptides, tannins, and saponins have been isolated from various hawthorn tissues [45]. Due to this chemical diversity, hawthorn shows considerable promise as a source for novel lead compounds in drug discovery and as a therapeutic agent for preventing and treating cardiovascular, cerebrovascular, and digestive tract diseases, in addition to possessing anti-aging effects [46]. For instance, studies have found that hawthorn ethanol extract (CPE) can inhibit the aggregation and degradation of amyloid-beta (Aβ) [47]. Through separation and identification, compounds including crataeguslignan A and 4″-O-(8-guaiacylglycerol) buddlenol A were isolated as the active ingredients [19]. Amyloid deposits and neuronal fiber tangles are among the causes of Alzheimer’s disease. These findings indicated that this class of compounds may have potential applications in the treatment of Alzheimer’s disease. However, a large number of clinical studies are still needed to support the evidence.

## 5. Extraction Process for Functional Materials

The study summarized and discussed the significance of bioactive compounds in hawthorn. To conduct a more scientific analysis of its potential properties, it is necessary to extract these compounds from hawthorn. Currently, the primary extraction methods for preparing water-soluble dietary fiber, hawthorn polysaccharides, flavonoids, and other active functional components domestically and internationally include traditional methods (maceration, percolation, decoction, reflux). Advance approaches include pressurized solvent extraction, countercurrent extraction, supercritical fluid extraction, ultrasonic-assisted extraction, microwave-assisted extraction, ultra-high-pressure extraction, rapid solvent extraction, flash extraction, enzyme-assisted extraction, dual-phase extraction, fermentation, and combined methods. A comprehensive evaluation of water-based extraction methods determined that the 60 °C water leaching method was the optimal technique for extracting mixed active substances [48]. By optimizing the ultrasonic-assisted extraction process for flavonoids from hawthorn seeds, the optimal conditions were determined, such as an ultrasound temperature of 65 °C, an ultrasonic time of 37 min, an extraction temperature of 91 °C, an extraction time of 90 min, a solid–liquid ratio of 1:18, and 72% ethanol [49]. One of the methods includes an ionic liquid (IL)-based one-step micellar extraction procedure for extracting multiple polar compounds from hawthorn berries. Compared to traditional methods, this approach is simpler, more sensitive, environmentally friendly, and highly efficient [50]. It is a synergistically enhanced DES extraction technique using ultrasonic and resin in situ adsorption to extract phenolic compounds from hawthorn seeds. The results indicated that the process involving choline chloride and oxalic acid, along with non-resin XDA-1, facilitates the extraction of polyphenolic compounds [51]. A comparative analysis of different applications for extracting water-soluble dietary fiber (SDF) from hawthorn pomace was conducted. The results indicated that the microwave-assisted enzymatic method yielded the optimum SDF extraction rate [52].

## 6. Current Research on Hawthorn Processing Applications

**Figure 10:**
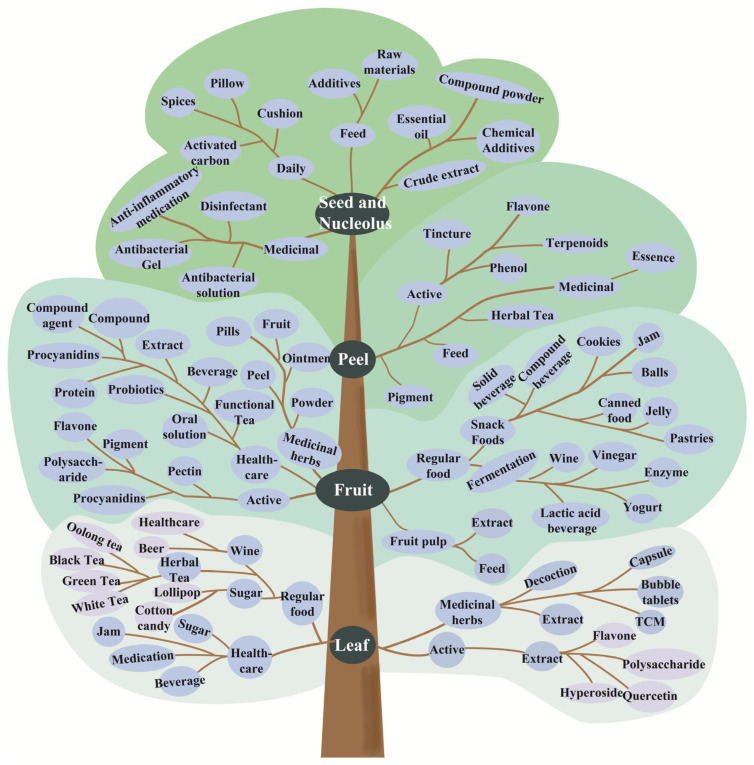
Hawthorn processing layout from different plant parts.

The hawthorn plant is edible. Its fruit is dried and pressed for extensive use in the pharmaceutical, food, and agro-industries sectors, including the production of common snacks and health products (Figure 10).

### 6.1. Hawthorn Leaf

Extensively researched for its potent antioxidant and cytoprotective properties, the often-overlooked hawthorn leaf byproduct can be made into a tea rich in active ingredients, such as polysaccharides, flavonoids, and triterpenes [53]. Polyphenols, the primary antioxidants in hawthorn, effectively scavenge superoxide anions [54]. Supported by correlation analyses, these properties make hawthorn leaf extracts promising for use in functional foods, nutritional supplements, and cosmetic or medical hygiene products [55]. Consequently, the extract has been approved by the China Food and Drug Administration for treating heart disease and hyperlipidemia [56].

### 6.2. Hawthorn Peel

Hawthorn peel, though often discarded or dried for infusions due to its sour and astringent taste, is a rich source of bioactive compounds, such as polyphenols, flavonoids, and triterpenoids [57]. Studies indicated that these compounds can be co-extracted, and the peel contains significantly higher levels of polyphenols and flavonoids than fruit pulp, underscoring its promise as a raw material for industrial extraction and medicinal use [58].

### 6.3. Hawthorn Fruit

The hawthorn fruit serves as the base for over 100 distinct products. These encompass everything from primary processed forms, such as dried, powdered, and pulped hawthorn to traditional delicacies, beverages, and fermented foods. Its applications extend to the pharmaceutical sector and even to other industrial uses like animal feed and feed additives.

#### 6.3.1. Traditional Hawthorn Products

This study systematically studied the packaging, sweeteners, gelling agents, and process optimization for apricot and rose jam composite fruit leather, providing a theoretical basis for its production and storage [59]. A sucrose-free hawthorn fruit peel using oligoxylose and xylitol as sucrose substitutes was shown to prevent constipation [60]. The study also optimized the formula and microwave sugar infiltration process for low-sugar hawthorn preserved fruit from multiple perspectives [61,62]. Furthermore, research on hawthorn cakes has primarily focused on the process of optimization and multi-dimensional formula innovation [63], exemplified by varieties, such as lily, persimmon, and red date hawthorn cakes.

#### 6.3.2. Hawthorn Juice Drinks and Fermented Hawthorn Products

The sour and astringent taste of hawthorn juice, caused by its rich nutrient profile, limits its consumer appeal and sales, making the development of diversified products a key priority. While techniques, such as high-pressure processing [64], are applied to improve the sensory profile of hawthorn juice, its combination with probiotic-rich yogurt presents another avenue for product development [65,66,67]. Yogurt is beneficial for its health benefits, including promoting intestinal health and calcium absorption. Remarkably, fermenting a mixture of hawthorn and yogurt yields a product with viable probiotic count greater (1.86 times) than standard yogurt [68,69]. The national promotion of “fruit-instead-of-grain” winemaking has driven growing interest and market demand for fruit wines, leading to increasingly in-depth research on hawthorn wine and fruit rich in fermentable sugar. Hawthorn wine is produced in various forms, such as hawthorn fruit wine [70], glutinous rice wine [71], brandy [72], distilled wine [73], health wine [74], and beer [75], respectively. Research has mainly focused on processing technology [73], fermentation strains [76], applications, and their impact on quality [77].

Studies indicated that vinegar, which is produced via acetic acid fermentation of alcohol, possesses a blood sugar-lowering effect. While hawthorn is nutritious, its unpalatable taste often leads to its use as a compound ingredient, fermented alongside other fruits to produce composite fruit vinegar [78]. Previous studies on hawthorn vinegar have mainly investigated its production process, aromatic compounds, and health efficacy. The fermentation process, driven by microorganisms like yeast, lactic acid bacteria, and acetic acid bacteria, generates various enzymes and bioactive compounds. Consequently, hawthorn vinegar is classified as a functional food, with purported benefits including regulating acid–base balance, enhancing metabolism, and promoting skin health. Hawthorn pomace, a byproduct of juicing rich in dietary fiber, represents a high-quality substrate for enzymatic preparation. Current research on hawthorn-derived enzymes has largely focused on fermentation methodologies and product quality assessment [79], encompassing specific investigations into mixed-strain fermentation [80], optimization of raw material ratios [81], fermentation process parameters [82], and antioxidant activities [83].

#### 6.3.3. Other Products

Hawthorn, a plant with a long history of medicinal and edible use, is a primary raw material in traditional Chinese medicine and modern health supplements [84,85], encapsulated in products such as hawthorn and danshen dispersible tablets and effervescent tablets. Although a waste product from food production, hawthorn residue has potential as a resource. For example, as a feed additive in aquaculture, it has been shown to improve lipid metabolism and enhance production performance in animals [86,87].

### 6.4. Hawthorn Kernel

Hawthorn kernels, when processed into powder, function as a valuable raw material. They can be used directly or as an auxiliary substance for the extraction of bioactive compounds, including total flavonoids and phenolic acids [88]. Their versatility allows for further development into a range of products spanning the pharmaceutical, food, and industrial sectors, such as natural medicines, food additives, activated carbon, and specialty oils [89,90]. GC-MS analysis of the main hawthorn seed extracts, obtained by dry distillation at different temperatures, revealed that the fraction collected at 211–230 °C exhibited the strongest antibacterial activity and optimum concentration of major compounds [91].

### 6.5. Current Status and Prospects of Hawthorn Industry Development

According to the available literature, there are thousands of hawthorn processing and sales companies in China, most of which are small- and medium-sized enterprises. Only a small proportion of companies are large, leading enterprises. Using whole-network big data, the top ten brands of hawthorn products have been selected based on multiple indices, such as brand value, word-of-mouth evaluation, sales volume, and industry recognition. These brands offer more than 30 different product forms, including hawthorn strips, hawthorn slices, hawthorn chicken gizzard slices, and hawthorn tea. Hawthorn processing and sales companies in China are unevenly distributed, heavily influenced by the location of hawthorn cultivation. These companies are primarily located in the Hebei, Shandong, Henan, and Shanxi provinces, as well as major municipalities like Beijing and Shanghai. The production of hawthorn products also includes specialized items like hawthorn powder, paste, and dried fruit mainly produced by pharmaceutical companies, while food items, such as hawthorn cakes, biscuits, and candies are primarily handled by the food processing industry.

A significant challenge is the lack of leading companies and advanced processing for different hawthorn products. Items like hawthorn shreds, honey, and jam have few top-ten-ranked producers and are typically only primary processed, indicating limited technological development. Simple processing technologies, such as drying, boiling, and distillation are low in technical complexity and low in product-added value. Consequently, the market exhibits significant disparity among enterprises, with a clear divide between those producing a diverse product line and those limited to only one or two varieties. Guangxi is a leading production region for large-fruit hawthorn in China, with a cultivation area exceeding 10,000 ha and an annual output of approximately 60,000 tons. The local industry includes ten major agroprocessing plants that produce a variety of products, such as hawthorn cakes, slices, wine, and vinegar. Even so, Guangxi has not been considered in the top ten brands. This shows that the evaluation, sales, and industry recognition of hawthorn enterprises in Guangxi still need to be improved globally.

## 7. Conclusions and Future Outlook

This paper compiled the distribution of Chinese hawthorn germplasm, along with its nutritional and functional components, processing strategies, and industrial status. Furthermore, we present a novel taxonomic tree of its germplasm resources, offering a clearer systematic understanding of the plant. Hawthorn is an important medicinal and edible plant, valued for its unique flavor, rich nutritional profile, and abundance of bioactive substances. Researchers have analyzed its nutrients, verified its health benefits, and optimized processing approaches. Consequently, entrepreneurs have developed a diverse range of hawthorn products, spanning from primary foods and refined extracts to chemical and pharmaceutical applications. Analysis of hawthorn enterprise landscape indicates an industry characterized by limited scale and uneven regional development. China’s Guangxi province, which is a key producer of large-fruited hawthorn, is notably underrepresented among the industry’s top-tier brands. For Guangxi’s hawthorn enterprises to gain a competitive edge, significant improvements are required in different areas, including brand value, market reputation, sales volume, industry standing, brand heritage, international footprint, and accolades.

The world hosts a rich diversity of hawthorn species with complex phylogenetic relationships. Although China is a key center of origin for the genus, the evolutionary history of these plants remains controversial and requires further study. Furthermore, the establishment of core germplasm collections and molecular identification of local varieties are still limited. To support the conservation, evaluation, and breeding resources of hawthorn, molecular analyses of genetic diversity are urgently needed. These analyses should employ techniques, such as morphology, molecular markers, and simplified genome sequencing. Furthermore, research has confirmed that the pharmacological activity of hawthorn is primarily attributed to flavonoids like quercetin and hyperoside, as well as phenolic acids, which are often used as international quality control standards. However, the Chinese pharmacopeia uses only organic acids as indicators, which fails to fully reflect the active ingredients. Therefore, incorporating flavonoids into the quality control system is crucial in years to come.

## Figures and Tables

**Table 1:** Classification and growth characteristics of Chinese *Crataegus* plants.

Genus and Species	Genus and Species Grouping	Species/Latin Name	Variant	Classification/Geographical Distribution	Growth Distribution Especially in China	Growth Environment	Plant Characteristics
*Crataegus* L. genus, *Maloideae* subfamily, Rosaceae family	Sect. *Pinnatifidae* Zabel (2 species)	*Crataegus pinnatifida* Bge.	*C. pinnatifida* var. major	Widely distributed species	Northeast China, Inner Mongolia, Hebei, Henan, Shanxi, Shaanxi, Shandong, Jiangsu, and Zhejiang in China, etc.; North Korea and Siberia	Grown on the edge of hillside forests and riverbank thickets, at an altitude of more than 100 m	The leaves are deeply or shallowly pinnately lobed, with veins extending to the apex and the division of the lobes; the inflorescence is slightly pubescent; the fruit is red; there are three to five small cores, with smooth inner surfaces on both sides.
			*C. pinnatifida* var. psilosa				
			*C. pinnatifida* var. geholensis				
		*Crataegus brettscheideri* Schneid.	/	Narrowly distributed species	Jilin and Liaoning in China; Leningrad, Moscow, Bryansk, Kiev, Almaty and other places in Russia	Grown in Changbai Mountain and other places in the central and northern parts of Northeast China	
	Sect. Henryanae Sarg. (3 species)	*Crataegus scabrifolia* (Franch.) Rehder	/	Mid-range distribution species	Yunnan, Guizhou, Guangxi, Sichuan	Sunny mountainous areas, stream banks, mixed woods, and forest edge thickets at an altitude of 1400–3000 m	Leaves are shallowly lobed or undivided, with veins extending to the tips of the lobes; inflorescences are glabrous; fruits are yellow or red; there are three to five small cores, with smooth inner surfaces.
		*Crataegus hupehensis* Sarg.	*Crataegus hupehensis* var. flavida	Widely distributed species	Hubei, Hunan, Anhui, Shanxi, Hebei, Jiangxi, Jiangsu, Zhejiang, Guangdong, Yunnan, Guizhou, Chongqing, Sichuan, Shaanxi, Gansu provinces in China	Grown in bushes on hillsides, at an altitude of 500–2000 m	
		*Crataegus shensiensis* Pojark	/	Narrowly distributed species	Shaanxi, Shanxi, Gansu	Grown on hillsides and mixed woods, at an altitude of 1100–1800 m	
	Sect. Cuneatae Rehd (2 species)	*Crataegus shandongensis* f.z. lI ET w.d.Peng	/	Narrowly distributed species	Shandong	Grown on hillsides, 500–700 m above sea level	Leaves are undivided or shallowly lobed, often cuneate at the base; inflorescences are slightly pubescent; fruit is red; three to five small cores, smooth on both sides.
		*Crataegus cuneata* Sieb. et Zucc.	*Crataegus cuneata* Sieb et Zucc var. *shangnanensis* L. Mao et T. C. Cui	Widely distributed species	Henan, Anhui, Jiangsu, Zhejiang, Fujian, Jiangxi, Hunan, Hubei, Shaanxi, Shanxi, Sichuan, Guizhou, Yunnan, Guangdong, Guangxi in China; Japan	Grown on sunny slopes, barren hills, stream banks, and thickets; 250–2000 m above sea level	
			*Crataegus cuneata* Sieb. et Zucc. var. longipedicellata M. C. Wang				
			*Crataegus cuneata* Sieb et Zucc f. pleniflora S.X. Qian				
	Sect. Tomentosae Sarg (3 species)	*Crataegus wilsonii* Sarg.	/	Mid-range distribution species	Hubei, Anhui, Jiangxi, Shanxi, Henan, Zhejiang, Guizhou, Chongqing, Sichuan, Shaanxi, Gansu, Tibet	Grown on hillsides, valleys and shady forests, at an altitude of 800–2500 m	The leaves are shallowly lobed, with veins extending to the tips of the lobes; the inflorescence is densely hairy; the fruit is red; there are two to three small cores with indentations on both sides of the inner surface.
		*Crataegus oresbia* W. W. Smith.	/	Narrowly distributed species	Yunnan, Guizhou, Sichuan in China	Grown in bushes on light slopes, at an altitude of 2500~3300 m	
		*Crataegus aurantia* Pojark.	/	Mid-range distribution species	Hebei, Inner Mongolia, Shanxi, Henan, Gansu	In mixed woods, forest edges, and thickets on hillsides, at an altitude of 1000–1800 m	
	Sect. Sanguineae Zabel (9 species)	*Crataegus maximowiczii* C. K. Schneid.	Ningan hawthorn *Crataegus maximowiczii* var. ninganensis	Mid-range distribution species	Heilongjiang, Jilin, Inner Mongolia, Hebei, Shanxi, Hubei, Sichuan, Shaanxi, Ningxia; Siberia, Sakhalin Island, North Korea, and Japan	In mixed woods or on the edge of forests, river banks, ditches, and roadsides, at an altitude of 200–1000 m	Leaves are shallowly to deeply lobed, with veins extending to the tips of the lobes; inflorescences are glabrous or slightly pubescent; fruits are red, yellow, or black; there are three to five small cores, with indentations or honeycomb-like holes on both sides of the inner surface.
		*Crataegus sanguinea* Pall.	/	Mid-range distribution species	Heilongjiang, Jilin, Liaoning, Inner Mongolia, Hebei, Shanxi, Henan, Guizhou, Sichuan, Xinjiang; Volga River Basin of Russia, Siberia, and Mongolia	In mixed woods on hillsides or beside rivers, at an altitude of 900~3000 m	
		*Crataegus dahurica* Koehne ex C. K. Schneid.		Mid-range distribution species	Heilongjiang, Jilin, Inner Mongolia, Hebei, Shanxi; Siberia and Mongolia	River valleys, hillsides, foothills, sand dunes, mixed woods, etc., with an altitude of 200–1800 m	
		*Crataegus chungtienensis* W. W. Smith.	/	Narrowly distributed species	Yunnan and Jiangxi	In mixed woods or bushes beside mountain streams, at an altitude of 2500–3500 m	
		*Crataegus kansuensis* E. H. Wilson	/	Mid-range distribution species	Hebei, Beijing, Shanxi, Henan, Shaanxi, Sichuan, Gansu, Qinghai, Ningxia, Xinjiang	In mixed woods, on shady hillsides, and beside ravines, at an altitude of 1000–3000 m	
		*Crataegus altaica* (Loudon) Lange	/	Narrowly distributed species	Xinjiang, Sichuan; European Russia, western lower Volga River, Siberia	Hillsides, under forests or beside rivers, at an altitude of 450–1900 m	
		*Crataegus remotilobata* Raikova ex Popov	/	Narrowly distributed species	Inner Mongolia, Shanxi, Xinjiang; Kyrgyzstan, Uzbekistan	Hillside ditch or roadside	
	Sect. Orientales Zabel	*Crataegus songarica* K. Koch	/	Narrowly distributed species	Xinjiang; Iran, Afghanistan, and Central Asia	River valleys, rocky hillsides or canyon bushes, 500–2700 m above sea level	Leaves are shallowly lobed or undivided, with veins extending to the tips of the lobes; inflorescences are glabrous; fruits are yellow or red; there are three to five small cores with smooth inner surfaces.
	/	*Crataegus beipiaogensis*	/	Narrowly distributed species	Liaoning	Hillside, 400 m above sea level	The leaves are deeply lobed, the inflorescence is hairy and yellow or red fruit; there are three to five small cores.
	/	*Crataegus laevicalyx*	/	Mid-range distribution species	Hebei, Beijing,	Foot of the mountain, sand slope, bush, 150–900 m above sea level	The leaves are undivided, with veins reaching the tips of the teeth; the inflorescence is hairless; the fruit is dark red, with a bluish-white powder on the surface; there are three to four small cores.
	/	*Crataegus wattiana* Hemsl. et Lace	/	Widely distributed species	Kunming, Yunnan (trial planting), Moganshan, Zhejiang (garden cultivation), Panzhihua, Sichuan (dry–hot valley trial)	Low and river banks	The leaves are shallowly lobed with veins reaching the tips of the lobes; orange-yellow to amber when ripe; three to five cores.
	/	*Crataegus jozana* Schneid	/	Narrowly distributed species	Jilin and Heilongjiang; northern East Asia (Sakhalin Island, Hokkaido, Japan, and the Russian Far East)	Mountain forest edge or shrub and, 500–1500 m above sea level	The leaves are shallowly lobed with veins extending to the top of the lobes; the fruit is dark green with white powder and two to three small cores.
	/	*Crataegus chlorosarca* Maxim	/	Mid-range distribution species	Liaoning, Jilin, Beijing, Shandong, Zhejiang; Sakhalin Island (Kankhalin Island), Kamchatka and even Japan	River valley area, 50–800 m above sea level	The leaves are shallowly lobed, with veins extending to the top of the lobes; hairless; bright green to yellow-green fruit (a few individuals have a slight red blush) with three to five small cores.
	/	*Crataegus tang-chungchangii* Matcalf	/	Mid-range distribution species	Fujian, Zhejiang, Jiangxi, Guangdong, etc.	Low-altitude mountains, forest edges, shrubs or roadsides; 200–1500 m above sea level	The leaves are shallowly lobed with veins extending to the top of the lobes; the inflorescence is glabrous or slightly pubescent; the fruit is red or orange-red; there are two to five small cores,
*Malus* genus, *Maloideae* subfamily, Rosaceae family	*Malus* *doumeri* (Bois) Chev	*C. pinnatifida* var. major (Large-fruited hawthorn)	/	Widely distributed species	Guizhou, Jiangxi, Zhejiang, Hunan, Guangdong, Guangxi, Yunnan; Taiwan	Deciduous, sparsely semi-evergreen shrubs or small trees	The fruit is large (80–200 g each), with oblate or pear-spherical. The skin is light yellow with a light red hue on the sun-facing side. The top of the fruit is concave, and the stalk is medium-long. The flesh is yellowish-white, thick, and dense. The fruit has six to eight ventricles and three to six seeds.

**Table 2:** Morphological differences in typical hawthorn varieties across regions.

Taste	Traits		North Crataegus (Bei Shanzha)	South Crataegus (Nan Shanzha)	Cantonese Crataegus (Guang Shanzha)
Fruit	Shape		Nearly spherical or pear-shaped	Nearly spherical or oblate	Nearly spherical
	Diameter (cm)		1.0~2.5	0.8~1.2	1.5~2.5
	Outer skin		Reddish brown to brownish red with small grayish white spots	Brown to brownish red, with fine wrinkles	Brownish red to brownish brown, with fine wrinkles
	Top part		There is a round deep depression at the top (commonly known as the pomegranate mouth), persistent calyx		Elongated cylindrical calyx
	Base part		With fruit stalk scars or residual fruit stalks, hairless		Depressed residual stem
	Carpel		Hard bone, paper-like		
	Taste		Sour, slightly sweet	Sour and astringent	Sour
Seeds	Number of seeds per chamber		One	One	Two
	Shape		Long withered petal-shaped	Withered petal-shaped	flat oval
	Size (cm)	long	0.7~1.2	0.5~0.6	0.8~1.4
		Width	0.3~0.6	0.3~0.5	0.5~0.7
	Surface View		Light yellow to light brown, with a deeper dorsal groove	Earthy yellow, shallow back groove	Light brown, shiny
	Texture		Hard bone, not easy to break		

## Data Availability

No new data generated.
